# Efficacy of OK-432 sclerotherapy for different types of lymphangiomas: a review and meta-analysis

**DOI:** 10.1016/j.bjorl.2023.03.007

**Published:** 2023-03-30

**Authors:** Jiali Sun, Changfeng Wang, Dan Song, Changhua Wu, Lei Guo

**Affiliations:** a Children's Hospital Affiliated to Shandong University, Department of Vascular Anomalies and Interventional Radiology, Jinan, China; b Jinan Children's Hospital, Department of Vascular Anomalies and Interventional Radiology, Jinan, China; c Shandong Provincial Clinical Research Center for Children's Health and Disease, Jinan, China

**Keywords:** Lymphangiomas, Sclerotherapy, OK-432, Meta-analysis, Efficacy

## Abstract

**Objective:**

This study aims to perform a meta-analysis to figure out the efficacy of OK-432 sclerotherapy between Macrocystic (MAC) lymphangiomas and Microcystic (MIC) lymphangiomas.

**Methods:**

We conducted a systematic review and meta-analysis to clarify the relationship between OK-432 and lymphangiomas. PubMed and ISI Web of Science were searched from inception to May 2022. Joanna Briggs Institute (JBI) manual was used to evaluate the risk of bias. We calculated pooled Relative Risks (RR) and 95% Confidence Interval (95% CI) using random effects model to evaluate the relations between OK-432 and lymphangiomas.

**Results:**

A total of 11 studies (including 352 cases) about OK-432 sclerotherapy for lymphangioma were included in the current meta-analyses. The results suggested that the efficacy of OK-432 was significantly in MAC lesions than in MIC (RR = 1.51, 95% CI 1.298–1.764), with significant moderate degrees of heterogeneity among 11 studies (I^2^ = 51.2%, *p* = 0.025). Subgroup analyses suggested that there was significant association in both retrospective studies (RR = 1.26, 95% CI 1.03–1.53) and classification (by 1 cm) (RR = 1.37, 95% CI 1.04–1.80) were associated with the efficacy of OK-432.

**Conclusion:**

To our knowledge, our study represents the first meta-analysis examining the efficacy of OK-432 in the treatment of different types of LMs. However, the regional differences and the age differences of the subjects are the main limitations of this study and should be avoided in further research. Our results suggested that OK-432 sclerotherapy for macrocystic lymphangiomas was more effective.

## Highlights

• OK-432 sclerotherapy for macrocystic lymphangiomas was more effective.
• This meta-analysis to verify whether the efficacy of OK-432 was related to classification for the first time.
• OK-432 should be used to therapy LMs with a lesion diameter greater than 1 cm.
• The classification basis is very important to the effect of sclerotherapy treatment.

## Introduction

Lymphangiomas (LMs) are kinds of benign, low-flow vascular malformation of the lymphatic system according to ISSVA (International Society for the Study of Vascular Anomalies)^1^ LMs may occur in any anatomic region of the body, but are particularly common in the neck and head (with incidence of 1.2–2.8 per 1000 live births),^2^ as well as axilla, mediastinum, groin, and retro-peritoneum.^3^, ^4^ The international incidence of LMs has been reported range from 1/6000 to 1/16,000 live births approximately.^5^ Besides, LMs represent 5%–6% of benign tumors in children.^6^, ^7^ Nowadays, LMs are still significant challenges to the diagnosis and treatment of interventional radiologists.

OK-432, also called picinanil, was originally developed in Japan as a chemotherapy agent.^8^, ^9^ The toxinproducing capacity of the bacterium is eliminated and its anticancer properties are strengthened after exposure to benzylpenicillin and heat treatment.^10^ Previous studies have shown that OK-432 is effective in treating patients with various cystic diseases, including ranula, salivary mucocele, auricular hematoma, thyroglossal duct cyst and other diseases.^9^, ^11^, ^12^, ^13^ And over the past 30 years, intracapsular sclerotherapy has become the most standard therapy for LMs.^14^, ^15^

Recent studies also suggested that the sclerotherapy effect of OK-432 may be related to the classification of LMs.^16^ Therefore, on the basis of reviewing previous studies, we used meta-analysis to verify whether the efficacy of OK-432 was related to classification for the first time.

## Methods

### Literature and search strategy

Two researchers independently searched the PubMed and ISI Web of Science databases from inception to May 2022 for related published studies. The literature search was limited to the English language. Index terms we used to search the indicate databases were ([lymphangioma] OR [lymphatic malformations] OR [LM] OR [LMs] OR [angiolymphoid]) AND ([OK-432] OR [Picibanil] OR [Sapylin]). Secondary references included in these literatures were also recruited. If more than one paper was published on the same cohort, only the study with the largest sample size was included.

### Study identification and selection

First papers without detailed data and duplicates in terms of OK-432 and LMs were excluded. Two reviewers independently assessed the articles for compliance with the inclusion criteria and resolved discrepancies by discussion until agreement was reached. Inclusion and exclusion criteria were shown in Table 1.

**Table 1:** Inclusion and exclusion criteria.

**Inclusion criteria**	(1) Evaluation of the efficacy of OK-432 on LMs.
	(2) Using descriptive study, case control study, cohort study, or randomized clinical trial design.
	(3) Research classify LMs into three or two types.
	(4) Containing complete data information.

**Exclusion criteria**	(1) Lymphangiomas were not classified according to lesions size or were unclassified.
	(2) Evaluation of efficacy between LMs and other sclerotherapy.
	(3) Studies of mechanisms based on genes or proteins.
	(4) Case reports, posters, guidelines, reviews, letters and meeting abstracts.

### Data extraction

**Figure 1:**
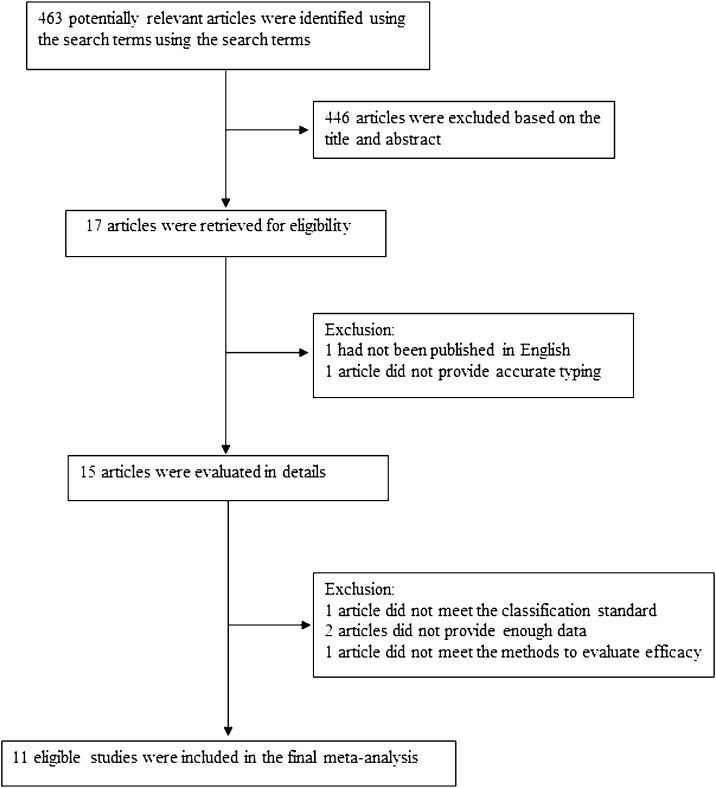
Flowchart of the study selection process.

The following information was extracted from each study: (1) Name of the first author; (2) Year of publication; (3) Country where study was done; (4) Sample size of the study; (5) Age range of the study population; (6) Number of different outcomes after injection of drugs for different types of lymphangiomas, mainly including number of effective or ineffective; (7) Number of males and females; (8) Number of cases with effective treatment; (9) Methods of diagnosis and evaluation and (10) Definition of classification. The classification of lymphangiomas was according to ISSVA. If there was discordance among the two independent researchers for one study, its eligibility was decided by the 3rd investigator. 11 publications^17^, ^18^, ^19^, ^20^, ^21^, ^22^, ^23^, ^24^, ^25^, ^26^, ^27^ with 352 patients were comprised. Detailed information about flowchart of the study selection process was shown in Fig. 1.

### Assessment of methodological quality

Preferred Reporting Items for Systematic Reviews and Meta-Analyses (PRISMA) Statement guidelines were used to report the results of this systematic review. Two independent reviewers used the Joanna Briggs Institute (JBI) Reviewers Manual to evaluate the bias risk of the 11 articles included in this review until consensus was reached.^28^, ^29^

### Statistical analysis

Heterogeneity was assessed by the Q-test and the I^2^ statistic.^30^ The fixed effects model was used when I^2^ value was greater than 50%. Otherwise, the random effects model was used. Sensitivity analysis was performed to further explore the source of heterogeneity. Publication bias was assessed by Begg’s test^31^ and Egger’s test^32^ and visually assessed by funnel plot; *p* < 0.05 was considered statistically significant. All the statistical analyses were conducted using STATA version 14 (StataCorp LP, College Station, TX, USA).

## Results

### Study characteristics (Table 2)

We included 11 associated studies from 11 publications in the current meta-analysis. All of them reported the results of OK-432 in the treatment of LMs. The subjects ranged in age from newborn to 78 years old. All the lesions were diagnosed clinically and with ultrasound, Computed Tomography (CT) or Magnetic Resonance Imaging (MRI), as well as interventional radiology examinations and classified according to the size of the cysts based on the radiological appearance. 6 publications^17^, ^18^, ^19^, ^20^, ^21^ classified Macrocystic (MAC) lesions MAC with a diameter greater than 2 cm, Microcystic (MIC) with less than 2 cm or mixed when both large and small cysts were present, with 156 participants. 5 publications^22^, ^23^, ^24^, ^25^, ^26^, ^27^ defined MAC with a diameter more than 1 cm, MIC with less than 1 cm and mixed, with 225 participants. All of the 11 studies were conducted in both male and female (Table 2).

**Table 2:** Characteristics of studies include in the meta-analysis of the association between OK-432 and lymphangiomas.

Study	Publish year	Country	Study design	Sample size	Age	Gender (F/M)	Number of effective		Diagnosis methods	Classification definition
							MAC	MIC		
Greinwald et al.	1999	American	Prospective study	12	1m^a^ to 94m	2/10	4	2	MRI/CT	MAC>2
										MIX<2
Giguere et al.	2002	American	Prospective study	29	6m to 18y^b^	12/17	18	1	MRI/CT/Medical photography	MAC>2
										MIC(MIX)<2
Rautio et al.	2003	Finland	Retrospective study	14	10 m to 42y	6/8	7	4	MRI/CT/ Ultrasound	MAC>2
										MIC(MIX)<2
Weitz-Tuoretmaa et al.	2014	Finland	Retrospective study	36	1 m to 47 y	16/20	22	2	Clinical/MRI	MAC>2
										MIC (MIX)<2
Malic et al.	2017	Canada	Retrospective study	27	Unmentioned	12/15	14	5	Interventional	MAC>2
									Radiology	MIC (MIX)<2
Cantú-Reyes et al.	2018	Mexico	Retrospective study	26	Unmentioned	16/10	16	5	Radiological	MAC>2
							MAC	MIC		MIC(MIX)<2
Claesson et al.	2002	Sweden	Prospective study	32	2 m to 64y	23/9	17	9	MRT/CT/ Ultrasound	MAC>1
										MIC (MIX)<1
Luzzatto et al.	2005	Italy	Retrospective study	27	Newborn to 14y	8/19	12	5	Ultrasound	MAC>1
										MIC (MIX)<1
Luzzatto et al.	2000	Italy	Retrospective study	15	Newborn to 15y	12/3	7	3	Ultrasound/MRI/ CT	MAC>1
										MIC (MIX)<1
Ghaffarpour et al.	2015	Sweden	Retrospective study	131	2m to 78y	74/57	25	67	Ultrasound/MRI	MAC>1
										MIC (MIX)<1
Gilony et al.	2012	Israel	Retrospective study	20	5 m to 10 y	Unmentioned	13	6	MRI	MAC>1
										MIC (MIX)<1

PS: ^a^ Month; ^b^ Year.

### Results of meta-analysis

**Figure 2:**
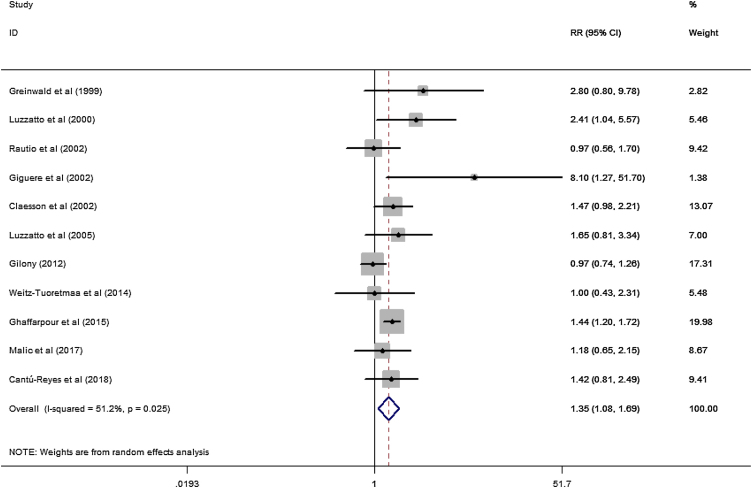
Forest plots of the summary Relative Risks (RR) with corresponding 95% CI for the association between efficacy of OK-432 and lymphangiomas.

**Figure 3:**
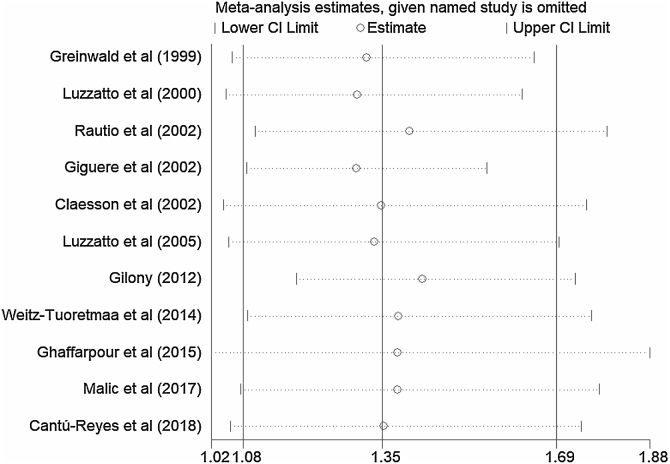
Sensitivity analysis of the pooled Relative Risks (RR) ranged from 1.30 (95% CI 1.09‒1.56) to 1.43 (95% CI 1.19‒1.73). No study had a significant impact on the total combined results.

**Figure 4:**
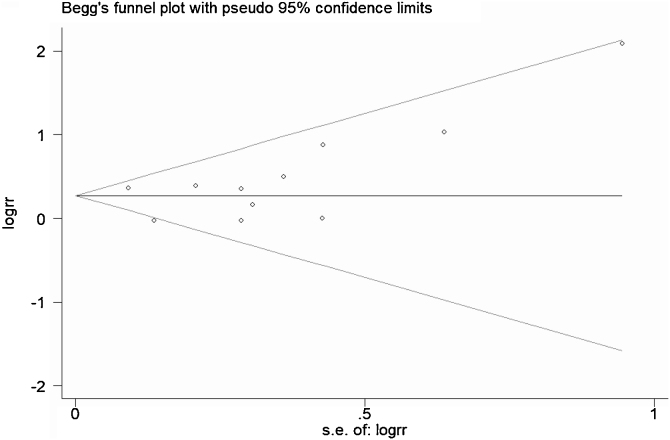
Egger’s test suggested that there was no publication bias.

**Figure 5:**
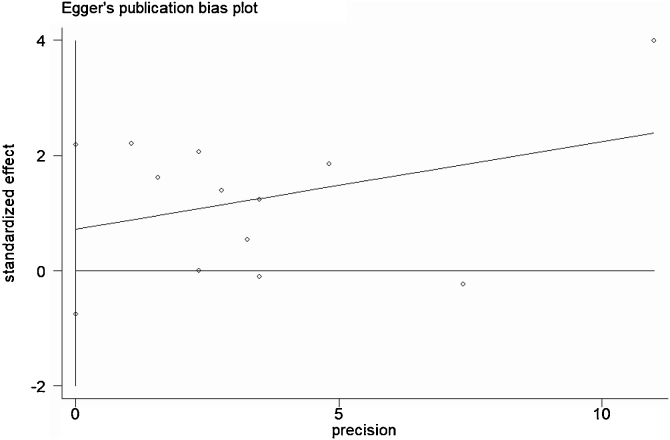
Begg’s test suggested that there was no publication bias.

**Figure 6:**
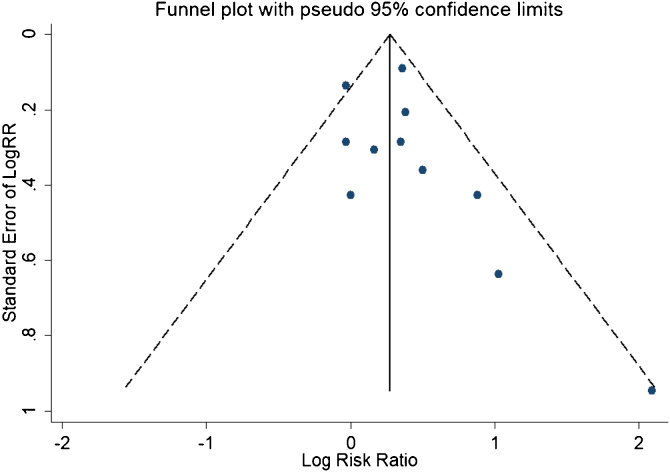
Funnel plots for detection of publication bias.

A total of 11 studies (including 352 cases) were included in the meta-analysis of the efficacy after sclerotherapy. The results suggested that OK-432 was significantly effective in treating large cystic LMs when compared with small cystic (RR = 1.51, 95% CI 1.298–1.764), with significant evidence of heterogeneity among 11 studies (I^2^ = 51.2%, *p* = 0.025), so we used a random effects model (Fig. 2). The result was stable after sensitivity analysis, the pooled RR (95% CI) ranging from 1.30 (1.09–1.56) to 1.43 (1.19–1.73) (Fig. 3). There was no evidence of publication bias with Egger’s test (*p* = 0.297, 95% CI −0.751 to 2.191) (Fig. 4) or with Begg’s test (*p* = 0.102) (Fig. 5). The distribution of funnel plots was symmetrical (Fig. 6). The results of JBI assessment showed that the quality of the included literature ranged from 6 to 8 points, which was consistent with the results of our meta-analysis.

To explore potential sources of moderate heterogeneity across studies and to examine the impact on final summary estimates, we conducted a series of subgroup analyses according to study design (prospective studies or retrospective studies) and the definition of classification (the classification was bounded by 1 cm in diameter or 2 cm). In the subgroup analyses, the association was significant in retrospective studies (RR = 1.26, 95% CI 1.03–1.54) (Supplementary Fig. 1). What’s more, the significant association remained for 5 studies which defined diameter greater than 1 cm (RR = 1.37, 95% CI 1.04–1.80) (Supplementary Fig. 2).

## Discussion

To date, many studies have investigated the association between OK-432 and sclerotherapy outcome of LMs.^17^, ^18^, ^19^, ^20^, ^21^, ^22^, ^23^, ^24^, ^25^, ^26^, ^27^ The researchers used prospective or retrospective studies to demonstrate the clinical efficacy of OK-432 in the treatment of LMs. But none of them has indicated which type of LMs is better treated with OK-432. However, the results have been inconsistent. Reyes et al. and Claesson et al. suggested OK-432 probed to be an effective treatment for LMs. But based on the findings, they concluded that the effect of sclerotherapy was independent of size, anatomical position or classification.^22^, ^23^ Some researcher even considered OK-432 unsuitable for sclerotherapy of abdominal lymphatic malformations because of the difficulty in reducing swelling after injection and the unclear effect on surrounding tissues.^33^ However, other researches believed OK-432 sclerotherapy to be a safe and effective treatment with a long lasting effect in the management of macrocystic LMs.^20^, ^21^, ^24^ The discrepancy might be due to the limited statistical power for most included cases. In the present meta-analysis, we confirmed the positive association between OK-432 and macrocystic LMs.

LMs are rare congenital malformation of lymphatic system.^34^ Sudden enlargement of the lesions usually signifies either infection or haemorrhage.^34^, ^35^ LMs have been proved mainly affecting children less than 1 year of age.^2^ Previous researches have confirmed the enlarged lymphatic vessels were covered with mural cells in lymphatic malformations.^36^ Meanwhile, experiments in molecular biology proved that Human Dermal Lymphatic Endothelial Cells (HDLECs) were in a mesenchymal status. But HDLECs lost their mesenchymal status after OK-432 treatment. In view of this, they suggested that the mechanism of OK-432 sclerotherapy may be that decreased LECs mesenchymal state may lead to vascular contraction.^36^ In another study, researchers declared that antigen-presenting cells and Toll-Like Receptors (TLR) seem to play a dominant role in the working mechanism of OK-432.^37^ Wiegand et al. pointed intracystic levels of interleukin (IL-6, IL-2R, tumor necrosis factor-α) were elevated after injection OK-432. They suggested that OK-432 can alter the level of interleukin which may be involved in the pathogenesis of LMs.^38^ The latest research reported that Genotype-Adjusted Variant Allele Fractions (GVAFs) are significantly higher in LMs and can activate PI3K to some extent, which can also make patients show more severe clinical symptoms.^39^

However, several limitations should be considered. First, the included studies were mainly conducted in Europe and America. Our findings might not be suitable to be extended to populations of other countries, such as in Australia. Second, there were age differences for the studies of first time received sclerotherapy. Third, the combination of microcystic and mixed type may have an impact on the judgment of efficacy.

## Conclusion

In conclusion, the current meta-analysis suggested that the efficacy of OK-432 is more effective in treating macrocystic LMs than microcystic LMs. The classification basis is very important to the effect of sclerotherapy treatment. Therefore, we suggest that OK-432 should be used to therapy LMs with a lesion diameter greater than 1 cm.

## Funding

This study is supported by Science and Technology Program of Jinan Municipal Health Commission (2022-2-144), Expression and clinical significance of IFN-γ in lymphatic malformation). Clinical Medical Science and Technology Innovation Program of JiNan science & Technology Bureau (202134070).

## Conflicts of interest

The authors declare no conflicts of interest.
