# Supplementary material for: Efficacy of OK-432 sclerotherapy for different types of lymphangiomas: a review and meta-analysis
Source: Braz J Otorhinolaryngol. 2023 Mar 30;89(4):101270. doi: 10.1016/j.bjorl.2023.03.007 (PMC10300298; doi:10.1016/j.bjorl.2023.03.007)
Supplement: Supplementary file 1 [file mmc1.doc]

**BJORL-D-23-00007_ Supplementary Material**

**Supplementary Figure 1** Subgroup analysis based on different design types with Relative Risks (RR) with corresponding 95% CI for the association between efficacy of OK-432 and lymphangiomas.


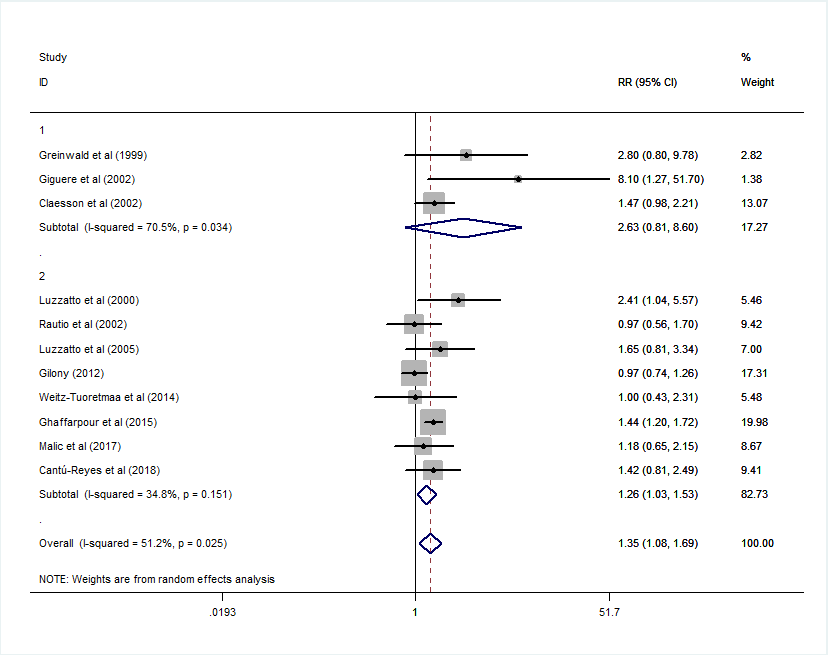


**Supplementary Figure 2** Subgroup analysis based on different classification definition with Relative Risks (RR) with corresponding 95% CI for the association between efficacy of OK-432 and lymphangiomas.

**
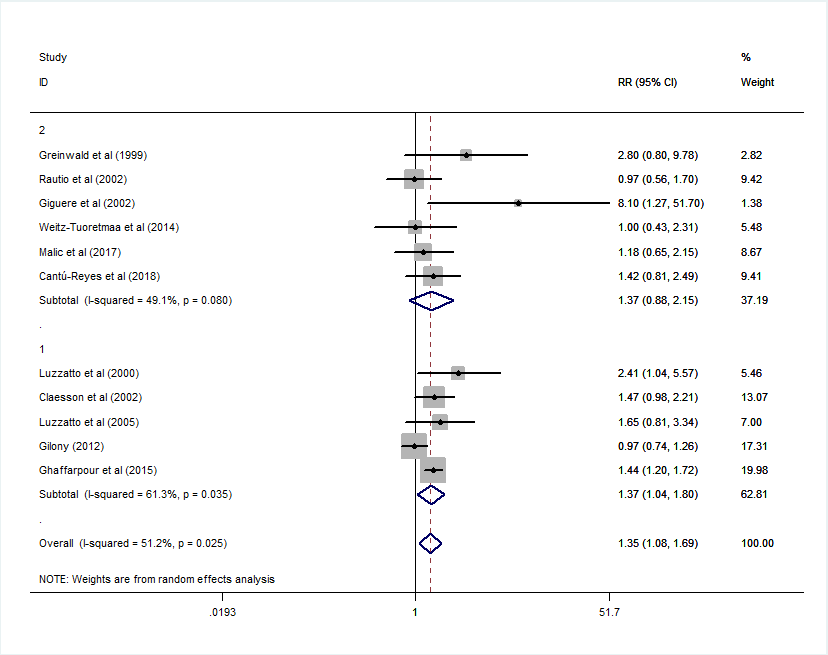
**
